# Supplementary figures and images for: Antibiotic persistence of intracellular Brucella abortus
Source: PLoS Negl Trop Dis. 2022 Jul 26;16(7):e0010635. doi: 10.1371/journal.pntd.0010635 (PMC9355222; doi:10.1371/journal.pntd.0010635)

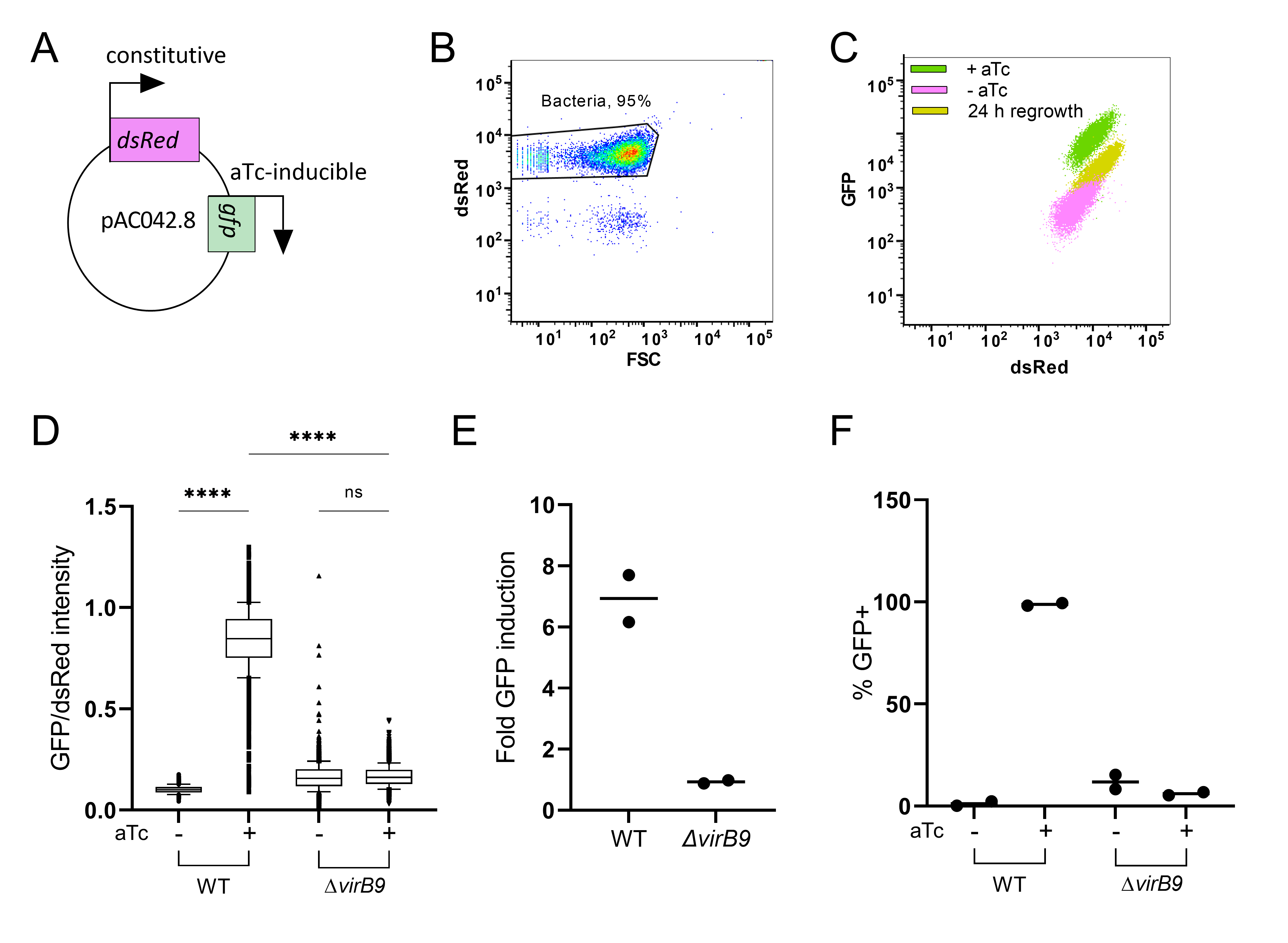

Supplement: S1 Fig — (A) Schematic representation of plasmid pAC042.8. Bacteria carrying pAC042.8 constitutively express dsRed and express GFP under a tetracycline-inducible system. (B) Flow-cytometric identification of the bacterial population grown till the exponential phase based on its dsRed fluorescence and forward-scatter (FSC) property. (C) Flow-cytometric detection of the GFP fluorescence in the bacterial population grown till exponential phase (magenta), after the addition of the inducer (green) and 24 h after removal of the inducer (yellow). For (B) and (C), displayed is a representative dot blot of one experiment of at least 3 independent replicates. (D, E, F) RAW macrophages were infected with B. abortus pAC042.8 or B. abortus ΔvirB9 pAC042.8 for 27 h. Cells were treated with aTc (100 ng/ml) at 23 hpi for 4 h before fixation and imaging. (D) Box plot showing the size-independent response of the bacterial population to the inducer (**** p ≤ 0.0001; ns = non-significant, one-way ANOVA and Tukey’s multiple comparison test). (E) Fold induction of the bacterial population response to the inducer. Fold induction was calculated by dividing the median values of the induced population by the non-induced one between matching conditions. Each dot represents one independent replicate, the horizontal line represents the mean, n = 2. (F) Percentage of GFP+ infection sites. Horizontal bars represent the mean, n = 2. (TIF) [file pntd.0010635.s001.tif]

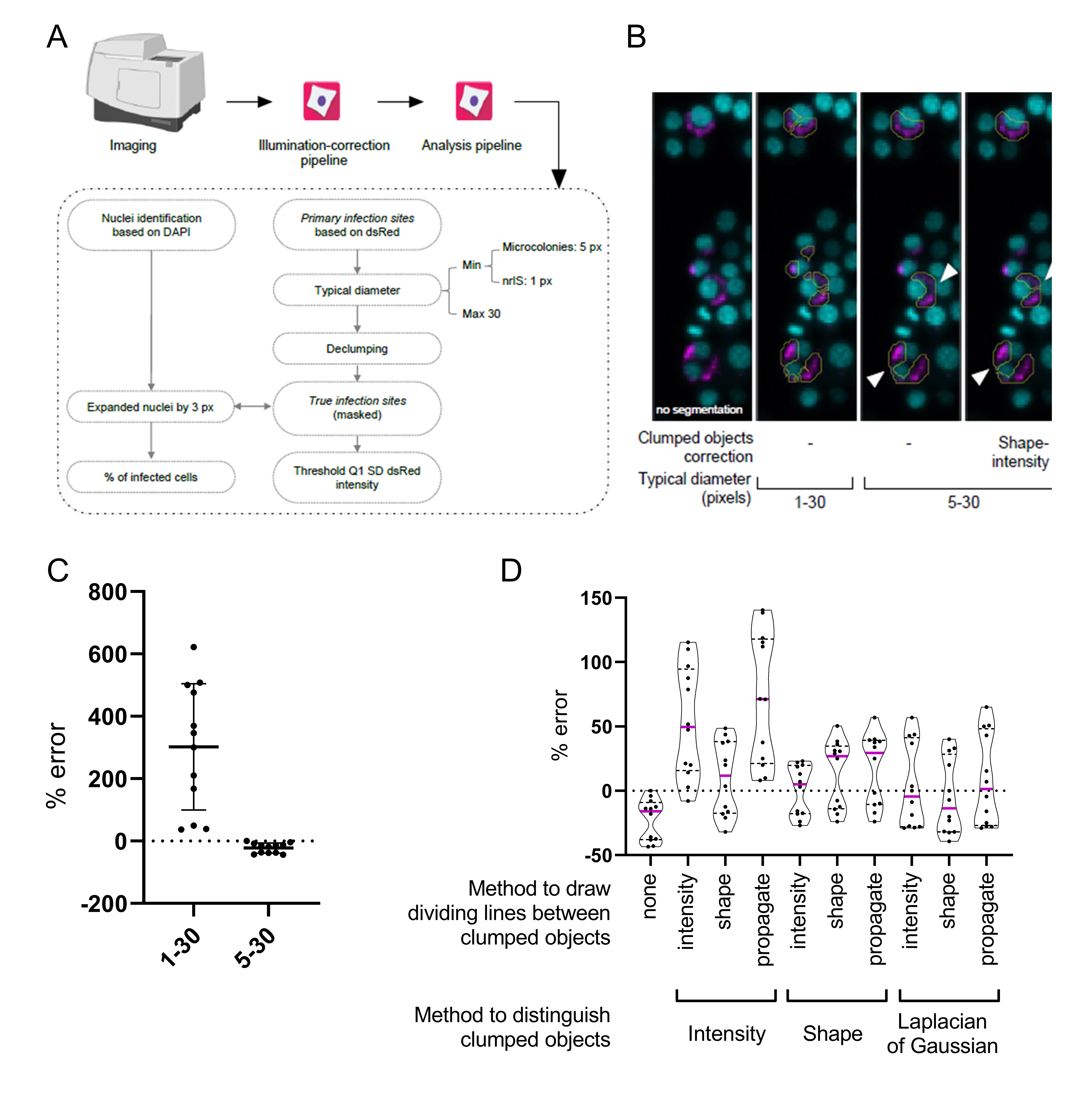

Supplement: S2 Fig — (A) Image analysis workflow for CellProfiler (https://cellprofiler.org) for the segmentation of nuclei and infection sites. The minimum typical diameter was set to different values in the pipelines to either identify non-replicative infection sites or microcolonies. Nuclei were expanded by 3 pixels and only infection sites residing inside the expanded nuclei were kept for the analysis. Created with BioRender.com. (B) Representative images from RAW264.7 macrophages infected for 27 h with B. abortus pAC042.8 constitutively expressing dsRed (magenta). Macrophage nuclei were stained with DAPI (cyan). Image analysis was performed with CellProfiler to segment nuclei and bacteria and to extract measurements. The left panel shows microcolonies segmentation with a typical diameter set between 1 and 30 pixels. The middle and right panels show microcolonies segmentation with a typical diameter between 5 and 30 pixels, without (middle) clumped-objects correction. The right panel shows microcolonies segmentation after identification of clumped objects based on the shape followed by the division of those based on the intensity. Arrows indicate examples of separation of merged microcolonies. (C) Percent error between manual count and CellProfiler count using a typical diameter between 1 and 30 or between 5 and 30. Data represent the mean ±SD, n = 10 (5 x 2 sites per experiment). (D) Violin plot showing the distribution of the percent error between manual count and CellProfiler count of microcolonies using several methods to distinguish and separate clumped objects. The pink line represents the median, dotted lines represent quartiles. n = 10 (5 x 2 sites per experiment). (TIF) [file pntd.0010635.s002.tif]

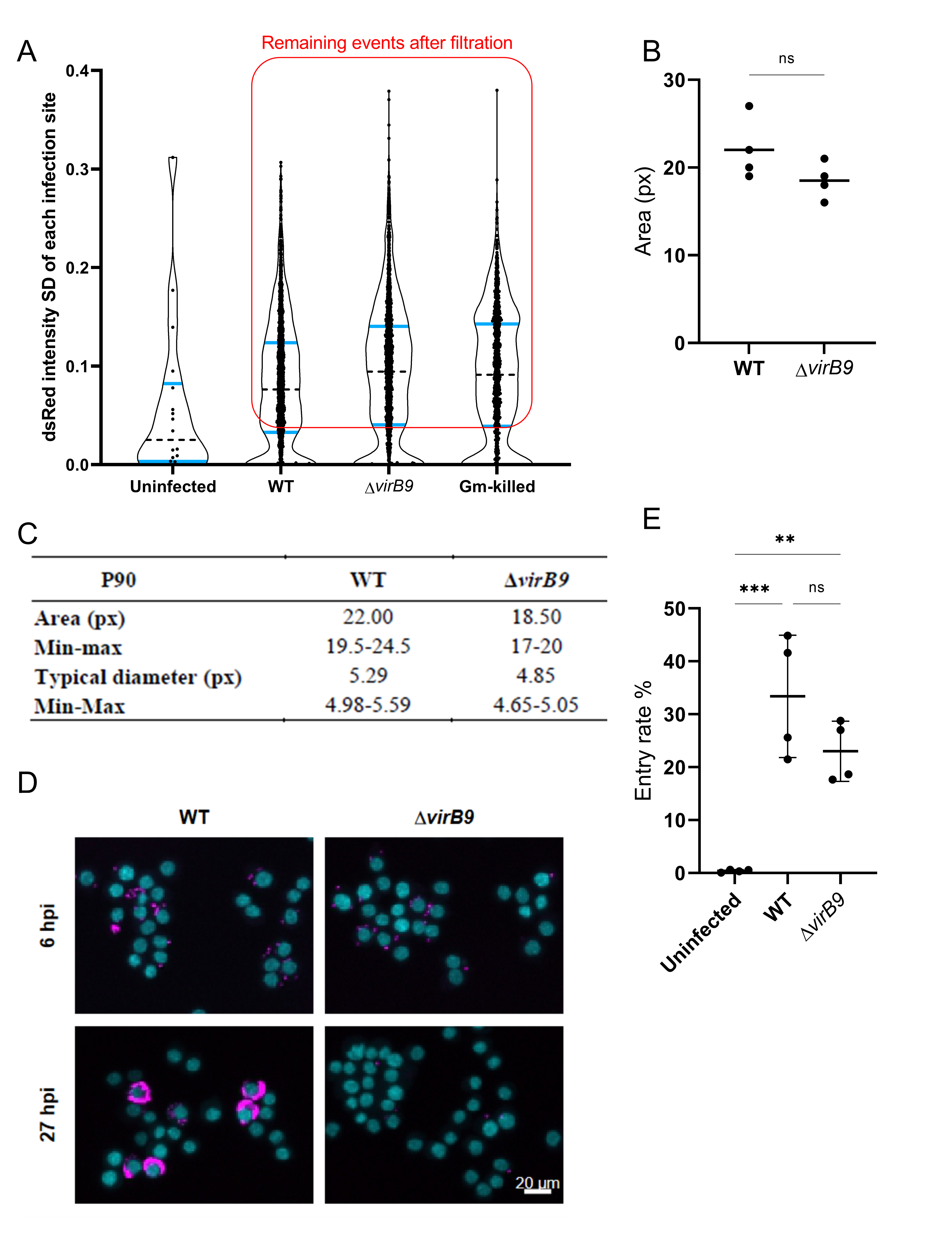

Supplement: S3 Fig — (A) Representative violin plot depicting the distribution of the standard deviation of the dsRed intensity for each infection site of B. abortus wild-type (WT), ΔvirB9 and wild-type killed with gentamicin for 24 h (GEN-killed) all carrying pAC042.8. Horizontal blue lines display first and third quartiles, horizontal black dotted line represents median. Red box shows remaining event after filtration with CellProfiler pipeline. n = 2 (B) Area of infection sites before intracellular replication. The 90th percentile (P90) of the measured area for each condition after 5 h infection. Horizontal lines represent the average, n = 4 (2 x 2 technical replicates, ns = non-significant with paired t-test). (C) Average area and typical diameter of infection sites of B. abortus wild-type (WT) and ΔvirB9 at 5 hpi. Table shows the equivalence between the measured area and the diameter of a circle using the formula dd = 2(√A/√π). Displayed are the averages from 2 technical replicates from 2 independent experiments. (D) Representative images from RAW264.7 infected for 6 h or 27 h with B. abortus WT or ΔvirB9. Bacteria constitutively expressed dsRed (magenta) and macrophage nuclei were stained with DAPI (cyan). (E) Entry rate from macrophages infected with B. abortus WT or B. abortus ΔvirB9 carrying plasmid pAC042.8 at 5 hpi. The entry was determined using the number of nuclei associated with at least one non-replicative infection site in the 3 pixels-expanded nuclei area. Horizontal lines show the average, n = 2 (2 x 2 technical replicates) (**p≤0.01, ***p≤0.001, ns = non-significant, one-way ANOVA and Tukey’s multiple comparison test). (TIF) [file pntd.0010635.s003.tif]

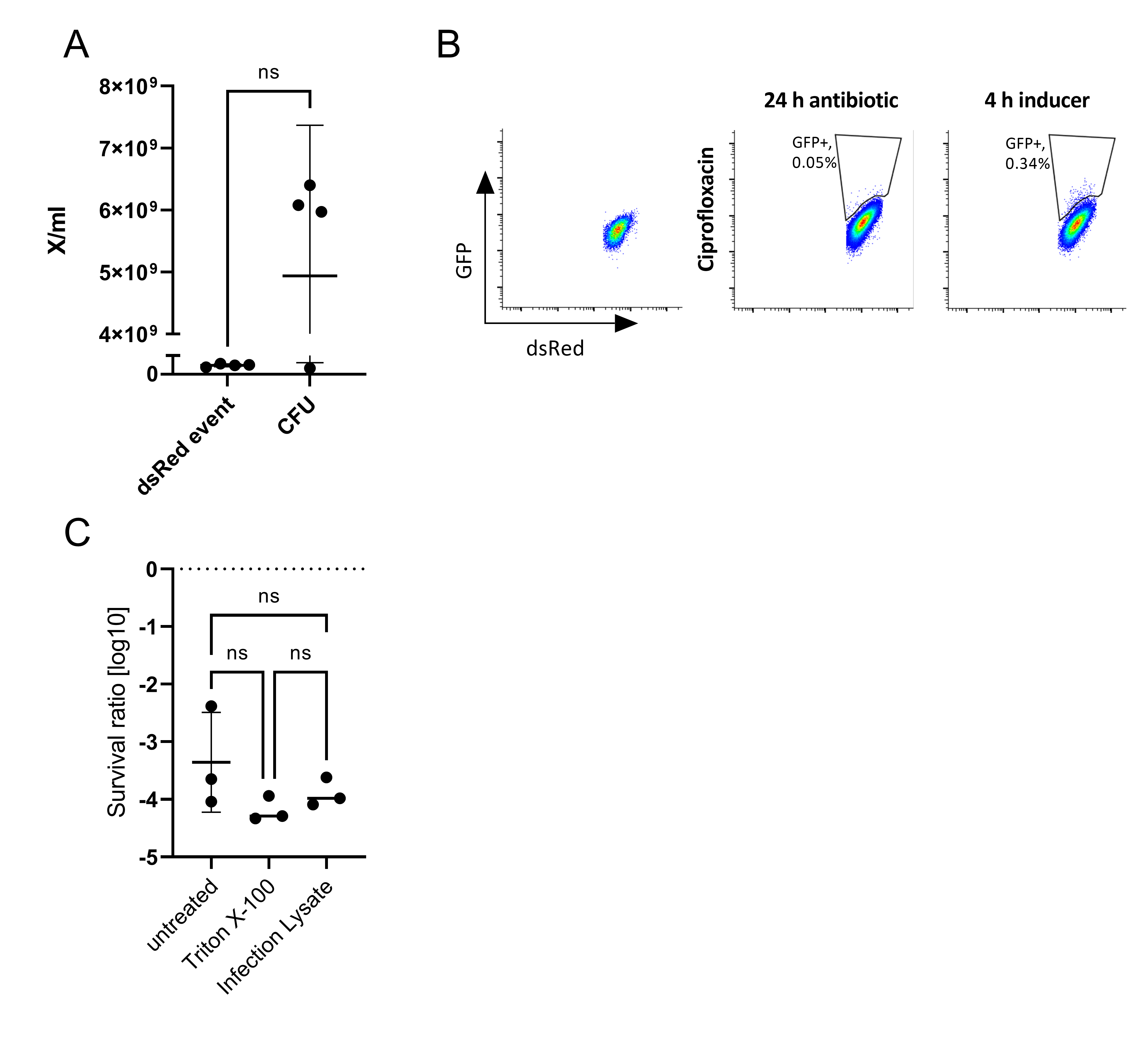

Supplement: S4 Fig — (A) Comparisons between CFU counts and flow cytometric counts using counting beads. Data represent the mean ± SD, n = 4 (ns = non-significant, paired t-test). (B) Flow-cytometric identification of the GFP+ population. Representative dot blots of one experiment of 3 independent replicates. (C) B. abortus was grown to mid exponential phase, macrophages were infected with B. abortus for 10 min, and B. abortus was incubated for 10 min in Triton X-100. After each step samples were washed, and one part of the sample was used to enumerate CFU/ml before ciprofloxacin treatment. Another part of each sample was subcultured in TSB plus ciprofloxacin and after 24 h CFU/ml were enumerated. The survival ratio was calculated by division of CFUs recovered before by CFUs recovered after ciprofloxacin treatment. (ns = non-significant, one-way ANOVA and Tukey’s multiple comparison test). (TIF) [file pntd.0010635.s004.tif]
