# Supplementary material for: Antibiotic persistence of intracellular Brucella abortus
Source: PLoS Negl Trop Dis. 2022 Jul 26;16(7):e0010635. doi: 10.1371/journal.pntd.0010635 (PMC9355222; doi:10.1371/journal.pntd.0010635)
Supplement: Attachment — Submitted filename: Point-by-Point Answer to the Reviewer Questions.docx [file pntd.0010635.s006.docx]

# Point-by-Point Answer to the Reviewer' Questions

## Reviewer #1 Summary and General Comments

In this manuscript, Mode and colleagues have investigated whether the worldwide bacterial pathogen *Brucella abortus* generates persisters in response to clinically relevant antibiotic treatments during its intracellular cycle in macrophages as the potential cellular basis for relapses associated with antibiotherapies against human brucellosis. Using a combination of advanced, automated microscopy-based analyses of dual fluorescent reporters and enumeration of viable bacteria, the authors report that i) *Brucella*’s intracellular niche in RAW264.7 cells provides partial protection against antibiotics, which do not fully inhibit bacterial replication, ii) a subpopulation of intracellular bacteria remains metabolically active and cultivable, iii) *Brucella* form persister cells in culture and intracellularly that fully retain their infection capabilities. Based in these findings, they conclude that *Brucella* intracellular persisters may constitute a population that mediates relapses associated with brucellosis, despite antibiotic therapies, and suggest changes in clinical practices.

This manuscript is clearly written and reports a well-designed and performed study that provides convincing evidence that a subpopulation of cultured or intracellular *B. abortus* display features consistent with a “persister” phenotype. As this study focuses on clinically relevant antibiotics and intracellular bacteria, it provides new information relevant to the treatment of brucellosis. I have a few concerns with the design of the microscopy-based detection of persisters and conclusions related to the intracellular source of persisters that need to be addressed, as detailed below.

We thank the reviewer for the constructive comments and suggestions. Hereafter is our answer the addressed points.

1. (A) By definition, persister cells should not grow or replicate intracellularly. It is therefore quite confusing that the fluorescence microscopy-based assay set up to identify intracellular persisters is based on the detection of foci of replicating bacteria inside cells during antibiotic treatments. These microcolonies clearly appear in the various micrographs provided as made of many bacteria (>50) so in no instance can they represent persister cells, which should show up as individual or small groups of bacteria (as shown for example in Fig. 3B). The authors define their assay as a readout of a population of intracellular bacteria that resist antibiotic treatment, which is technically correct, yet their replicative behaviour is inconsistent with the definition of persisters.

🡪 We understand that there seems to be some misunderstanding regarding our initial experimental approach. What we first assessed was the ability of antibiotics to prevent intracellular replication, using the decrease in intracellular replication as a proxy for antibiotic efficacy (Fig. 1 and 2).

We only then followed up by quantifying the proportion of intracellular *Brucella* still transcriptionally and translationally active under treatment conditions (Fig. 3) – and these are the data that primed us to look deeper into the observed “persister” phenotype. **Importantly, our data does not necessarily support bacterial growth of a subpopulation in contact with antibiotics** (see point 1 B just below).

(B) These remaining so-called microcolonies may reflect instead phenotypic variations in the macrophage population, where individual cells may not take up antibiotics as well (or exclude them efficiently) and provide as a result a permissive niche for bacterial replication, regardless of persisters formation.

🡪 The point raised here by the reviewer is also pertinent. Indeed, the single cell data (microscopy) presented in this manuscript does not allow to exclude that the microcolonies observed at high antibiotic concentration are the result of heterogeneous intracellular antibiotic concentration. Considering the reasonable doubt raised by the reviewer’s comment, we have clarified our conclusions as follows:

- Line 579-581 “The protective nature of *Brucella’s* intracellular state could also result – at least in part – from a possible phenotypic heterogeneity in the macrophage population in respect of antibiotic uptake and/or efflux.”

(C) As an example, Fig. 2E is presented to illustrate microcolonies in CIP-treated cells, but other cells in the field contain bacteria that display less replication and may represent true persisters. Yet, my understanding is that these were not counted in the analysis performed, or were they? This aspect of the study needs to be clarified.

🡪 As indicated above (point 1A), the experiments presented in Fig. 2 did not directly address the presence of persisters but “only” assessed the efficacy of antibiotics to inhibit intracellular replication in our macrophage infection model. Therefore, single dsRed positive bacteria (and/or small groups of bacteria) were not scored in the data presented in Fig. 2. We scored as “cells with intracellular replication” these containing bacterial cluster above a fixed size threshold i.e. microcolony, defined on the basis on the data presented in Fig. S3.

Importantly, the sole presence of dsRed signal in antibiotic treated macrophages was not considered as evidence for bacterial survival. In the data presented in Fig3 A-B we show that only a small fraction of the dsRed bacteria are actually transcriptionally and translationally active after antibiotic treatment.

2. A key part of the study is the demonstration of persisters formation in macrophages, which the authors associate with the protective environment of the intracellular niche and in particular the ER-derived replicative vacuole. However, the survival ratio remains relatively constant between 6, 24 and 48 h pi (Fig. 4D), while the ER-derived vacuole is not generated by 6 h pi in macrophages, even if bacteria start resuming growth (but not replication!) by then (Deghelt et al, 2014). This raises the question of the intracellular environment that promote persisters’ formation. The authors do not show whether persisters form in endosomal vacuoles (which are detectable throughout the whole cycle at frequencies consistent with persisters’ levels, even in the absence of antibiotics) or ER-derived vacuoles. In other words, does Brucella need to replicate in its ER-derived vacuole to form persisters? The authors make this claim but do not provide hard evidence for it. Is there an increased proportion of bacteria in endosomal (LAMP1+) vacuoles upon antibiotic treatment? This could be addressed by characterizing the intracellular niche of GFP+DsRed+ bacteria in antibiotic-treated cells.

🡪 There are different aspects to this interesting comment, and it seems that we need to clarify some of our statements as we feel that our conclusions may have been misleading.

**2.1 Regarding the influence of the intracellular trafficking stages on *Brucella* ability to survive antibiotic treatment.**

This is clearly an very interesting aspect, and we acknowledge that our data do not shed much light on this aspect. One cause of misunderstanding possibly lays in our use of “intracellular niche” to refer to “intracellular bacteria” without distinction of their sub-cellular compartmentation. A key point of our study is indeed the presence of “persisters” in *Brucella*-infected macrophages, without providing further characterization of the sub-cellular niche(s). To resolve this semantic ambiguity, we modified the manuscript as follows:

(Line 1) Title: Antibiotic persistence of intracellular *Brucella abortus*

- Line 25-28: “Combining microscopy of constitutive and induced reporters with classical CFU determination, we quantified the protective nature of the *Brucella* intracellular lifestyle to various antibiotics and the ability of *B.* *abortus* to persist *in cellulo* despite harsh antibiotic treatments.”
- Line 30-32: “We demonstrate that treatment of infected macrophages with antibiotics at recommended concentrations fails to fully prevent growth and persistence of *B. abortus in cellulo*, which may be explained by a protective nature of the intracellular niche(s).”
- Line 46-49: “Furthermore, we show that the macrophage intracellular lifestyle of *Brucella* has protective properties against recommended antibiotics as observed for other intracellular pathogens, highlighting the necessity to develop new infection models to assess antibiotic efficacy. ”
- Line 120-122: “Combining microscopy of constitutive and induced reporters with classical CFU determination, we quantified the protective nature of *Brucella´s* intracellular niche(s) to various antibiotics. ”
- Line 307-309: “Considering the proposed importance of the intracellular lifestyle of *Brucella* for its dissemination and maintenance within its hosts [26], we first investigated the influence of the intracellular state on the efficacy of different antibiotic treatments. ”
- Line 504-505: “Ciprofloxacin treatment in broth seemed to be more efficient compared to treatment of phagocytosed bacteria highlighting once again the protective nature of the intracellular niche(s).”
- Line 572: “A protective intracellular state”
- Line 573-574 “Our macrophage infection model unambiguously demonstrated the protective nature of *Brucella´s* ability to survive intracellularly.”
- Line 584-586: “Using an extracellular vs intracellular MIC comparison (Table 1), we determined that the protective effect of the intracellular state ranged from about 10-fold for rifampicin to roughly 50-fold for ciprofloxacin and doxycycline.”
- Line 593-594: “To overcome a potential protective effect of the *Brucella* intracellular niche(s), antibiotics could be loaded onto nanocarriers or into nanoparticles.”
- Line 629-632: “The combination of pathogen-specific factors and the nature of the intracellular niche(s) hence constitute additional layers of complexity when seeking for broad-range antimicrobials active *in cellulo* and eventually *in vivo.”*
- Line 656-660: “In that perspective, identifying the mechanisms underlying the formation of such sub-population(s), their associated physiological states, the exact nature of their intracellular niche(s), and assessing their possible involvement in the development of a systemic infection will constitute an important future research direction, as will be the investigation of persisters in clinical samples from human patients.”

Importantly, we show that reaching its replicative niche is **not** a pre-requisite for *Brucella* survival to antibiotic treatment (as demonstrated by the presence of “persisters” in macrophages already 6 h post infection, Fig. 4D, see also point 2.2). Similarly, we showed intracellular survival even when the macrophages were treated with antibiotics as early as 1 h post infection (Fig. 3).

The reviewer’s suggestion to quantify the effect of antibiotic treatment on the proportion of *Brucella* in LAMP1+ vacuoles, especially for those bacteria that showed the ability to express GFP under these conditions is an attractive idea. Considering the very low frequency of the described events (GFP+ DsRed+ events: appr. 2%, Fig. 3A and B), new methodologies will need to be developed to address these questions, which we feel is beyond the scope of this study.

**2.2 Regarding a link between intracellular growth and the observed survival to antibiotic treatment:**

We did not intend to claim that intracellular replication is required for *Brucella* to survive antibiotic treatment. Data acquired at 6 h post infection (Fig. 4D) – a pre-replicative stage in our infection model – already indicate that a fraction of the bacteria survive the antibiotic treatment when subjected to CIP after recovery from infected untreated macrophages. In our opinion, this demonstrates that replication is not required for the described phenomenon.

In the same experiment, we have measured an increase in the absolute number of intracellular “persisters” over time and a two log increase in the persister frequency. We have used this point as a hint of the possible *de novo* formation of persisters within the macrophage, and this in the absence of antibiotic pressure, as the antibiotic treatment was performed *ex cellulo*, on the bacteria recovered from lysed macrophages (Fig. 4D). It is however correct to say that we did not resolve in which intracellular compartment this formation happens. The advantage of this “ex-cellulo” procedure is that it minimizes the possible effect of antibiotic accessibility discussed under 1 (B) as the bacteria are recovered and freed from their intracellular niche before treatment.

Our hint that persisters *may* be formed during infection solely relayed on the before mentioned data. We acknowledge that the conclusion remains hypothetical, based on the possible alternative explanation and have adjusted the manuscript accordingly (see answer to point 1).

## Reviewer #1: Minor comments:

1. Fig. 3A: how much of the intracellular bacterial population turn GFP+ upon aTc induction in cells not treated with antibiotics? This could be a useful control to include.

🡪 The overall intracellular response to aTc induction in untreated cells was above 98% (vs. 0% in un-induced conditions), as depicted in Fig. S1F (23 h infection plus 4 h aTc induction, *B. abortus* WT). See also Fig. 3B for a visual example. For clarity, we also included the definition of the “infection sites” as “any dsRed+ object” in the legend of Fig. 3A.

2. Fig. 3B is important as it is the only visualization provided for individual or small group of metabolically active bacteria that fit the definition of persisters. I suggest the authors magnify the insets of the split DsRed and GFP channels to better show these particular events in CIP-treated cells.

🡪 We have implemented the reviewer’s suggestion in Fig. 3, as shown below, and completed the figure legend as follows: “Insets show split channels for dsRed (top) and GFP (bottom).” (Line 421-422)

3. line 84: should be “regimen”.

🡪 We have modified the manuscript accordingly

4. lines 444-445: based on most of the available literature, *Brucella* replication does not usually start by 6 h pi, as the replicative vacuoles are not formed by then. The increase growth plotted by the authors in Fig. 4D likely reflects a lack of time points between 6 and 24 h that would otherwise show that the net increase in bacterial numbers does not happen until later, i.e. 8-12 h pi depending on the cellular models. While this comment may appear nitpicky, it is actually important with respect to my main point 2.

🡪 Indeed, our 6 h time point aimed at addressing the pre-replicative state of the infection (Fig. 4D), whereas we chose 24 h post-infection to cover intracellular replication (Fig. 1, 2, 3, 4D). Please also see our answer to the main point 2 for further aspects related to this important point. For the sake of clarity, we modified the text as follows:

- Line 520-521: “Between 6 and 24 h the phagocytosed bacteria started to replicate leading to an increase of CFUs.”
- Line 523-525: “From 6 to 30 h the absolute number of persisters (Fig. 4D) increased although the persister ratio decreased since phagocytosed bacteria started to replicate.”

5. Fig. S3C: This figure exemplifies the efforts taken by the authors to best control their microscopic analysis of persisters, but it would really help to add when the analysis shown in table S3C was performed. Was it done at 6 h pi, when one can assume that the wild type and VirB-deficient population still behave similarly?

🡪 The analysis shown in table S3C was performed at 5 hpi, before onset of replication, to define the lower threshold to identify microcolonies at later time points for our CellProfiler pipeline. We adjusted the legend accordingly (Line 732). We also include the time reference for the data presented in Fig. S3E (5 hpi Line 738).

## Reviewer #2: General comment

This study aims to evaluate the antibiotic persistence of *Brucella* using a macrophage infection model. The authors use a *Brucella* strain carrying a dual reporter and evaluate bacterial persistence after antibiotic treatment by combining two techniques. (i) Microscopy of constitutive and induced reporters, and (ii) CFU determination. They conclude that antibiotic treatment fails to prevent the growth *B. abortus in cellulo*. They attribute the persistence of *Brucella* to the protective nature of the intracellular niche and the formation of intracellular persisters.

The authors explore a topic (barely explored experimentally) quite relevant to improving brucellosis treatment. The assays are well-validated, the results well explained, and the conclusions supported by their results.

🡪 We thank the reviewer for this positive feedback and the constructive comments.

## Reviewer #2: Minor concerns

1. Inhibition of bacterial replication in macrophages was only tested after 23 hours (Figure 2). Is there a reason for using this time point? Was bacterial replication evaluated for more extended periods? This could be relevant considering that *Brucella* treatments require multiple doses.

🡪 23 hpi lays within the usual time points to assess *Brucella* intracellular replication (20 – 30 hpi), which usually starts between 8 and 12 h post infection – and here served as a starting point for our study. We wanted to assess in a first experiment the influence of antibiotic treatment on intracellular replication of *Brucella* (Fig. 1 and 2). Longer incubation times with the established models are challenged by the replication of the RAW264.7 macrophages and would need further refinement of our experimental set-up and pipelines. Establishment of an *in vitro* model allowing long-term infection read-out surely constitutes an exciting challenge for future research.

2. Why were antibiotic persisters tested with a single antibiotic instead of a combination since it was shown that single treatments do not work?

🡪 This is a valid observation. For the inhibition of intracellular replication (Fig. 1 and 2) we also included clinically recommended antibiotic combinations. For our microscopic analysis of persisters *in cellulo*, however, we focused on ciprofloxacin (Fig. 3A), as doxycycline directly triggered the tetracycline-inducible promoter of our reporter system, turning every bacterium GFP positive before killing would occur. We included testing of different antibiotics (and combination) when assessing persistence of *Brucella* in treated macrophages by CFU plating (Fig. 3C).

3. Add a detailed statistical analysis section to the materials and methods section.

🡪 We have implemented this information in a separated method section (Line 295-301). We have also included a statistical analysis (ANOVA and t-Test) for the presented data, when relevant (Fig. 2F, 3A and C, S1D, E and F, S3B and E, S4A and C), with statistical tests now indicated in the corresponding figure legend.
